# Flushable Exo-Endodrainage: A Modified Palliative Approach to Non-Resectable Malignant Biliary Obstruction

**DOI:** 10.1155/1993/87181

**Published:** 1993

**Authors:** M. Stierer, H. Wasl, H. R. Rosen, A. P. Marczell, H. Spoula

**Affiliations:** 1 Hanusch Medical Center Department of Surgery Vienna Austria; 2 Hanusch Medical Center Department of Surgery Heinrich Collinstr 30 Vienna A-1140 Austria

## Abstract

The introduction of new imaging techniques has markedly improved the diagnosis of hepatobiliary disorders. Due to their anatomic situation, a substantial percentage of malignancies located near the hilus is not suitable for surgical management. We discuss an effective palliative intervention to relieve jaundice. In many instances drainage is a superior choice when biliodigestive anastomoses are not technically feasible and palliative resection carries a high complication rate.

We present an irrigatable exo-endodrainage method employing a modified port-a-cath system as a new alternative. In four patients, all older than 75 years, this system was implanted because of jaundice due to unresectable malignant stenosis of the extrahepatic bile duct. One patient (80 years old) died of pre-existing acute necrotizing pancreatitis, although hyperbilirubinemia was found to decrease on the 7th postoperative day. The other three patients showed complete normalization of their bilirubin levels and their port-a-cath systems remained open until their death (at 3 weeks, 6 months and 7 months respectively).


*HPB Surgery*, 1993, Vol. 7, pp. 25–32
Reprints available directly from the publisher
Photocopying permitted by license only
© 1993 Harwood Academic Publishers GmbH
Printed in the United States of America

### FLUSHABLE EXO-ENDODRAINAGE: A MODIFIED PALLIATIVE APPROACH TO NON-RESECTABLE MALIGNANT BILIARY OBSTRUCTION

M. STIERER, H. WASL, H.R. ROSEN, A.P. MARCZELL and H. SPOULA
*Hanusch Medical Center, Department of Surgery, Vienna, Austria*

*(Received 28 September 1992)*

The introduction of new imaging techniques has markedly improved the diagnosis of hepatobiliary disorders. Due to their anatomic situation, a substantial percentage of malignancies located near the hilus is not suitable for surgical management. We discuss an effective palliative intervention to relieve jaundice. In many instances drainage is a superior choice when biliodigestive anastomoses are not technically feasible and palliative resection carries a high complication rate.

We present an irrigatable exo-endodrainage method employing a modified port-a-cath system as a new alternative. In four patients, all older than 75 years, this system was implanted because of jaundice due to unresectable malignant stenosis of the extrahepatic bile duct. One patient (80 years old) died of pre-existing acute necrotizing pancreatitis, although hyperbilirubinemia was found to decrease on the 7th postoperative day. The other three patients showed complete normalization of their bilirubin levels and their port-a-cath systems remained open until their death (at 3 weeks, 6 months and 7 months respectively).

KEY WORDS: Non-resectable malignant biliary obstruction, jaundice, palliative surgery

### INTRODUCTION

Although primary carcinoma of the extrahepatic bile ducts are among the rarer forms of malignant tumors encountered in the gastrointestinal tract, incurable jaundice due to various causes (e.g. pancreatic, gall bladder, gastric carcinoma) is a common problem in abdominal surgery. Its prognosis is unfavorable[1,2] despite the improved diagnostic potential of ERCP, CT, sonography and angiography[2,3], as well as aggressive surgical procedures such as simultaneous removal of adjacent structures with vascular replacement and liver transplantation[4]. Especially with tumors of the hepatic porta and with Klatskin tumors[5], resection is difficult because of surgical-technical problems. Since the incidence of radical resection of tumors of the extrahepatic bile duct is approximately 30% [6], the remaining patients mainly require palliative drainage to remove the cardinal symptom, i.e. mechanical obstruction of the bile flow and its subsequent symptoms (jaundice, cholangitis, liver abscess, hepatic failure).

Palliative drainage in these situations comprises surgical and non-surgical modalities as illustrated in Table 1. These procedures are frequently associated with a number of complications that are either related to the procedure itself (hemorrhage, pneumo- and hematothorax, perforation) or to occlusion and dislocation of

Address correspondence to: M. Stierer, MD, Hanusch Medical Center, Department of Surgery, Heinrich Collinstr 30, A-1140 Vienna, Austria

**Table 1** Palliative surgical and non-surgical drainage modalities for non-resectable carcinoma of the extrahepatic bile duct

| | |
|---|---|
| *A. Surgical modalities* | |
| a. Biliodigestive anastomosis | |
| b. Internal drainage: 'lost' drain (7) | |
| U-tube (21) | |
| c. Combined external and internal drainage: | continuous (22) |
| | T-drainage (23) |
| | percutaneous endodrainage with plug (24) |
| *B. Non-surgical modalities* | |
| a. Percutaneous-transhepatic catheter drainage | |
| b. Percutaneous implantation of prosthesis | |
| c. Endoscopic implantation of prosthesis (stent, pig-tail) with/without sphincterotomy (25) | |
| d. Combined method of b. and c. | |

the drainage system[7,8]. Furthermore, some of the described drainage drainage methods are accompanied by technical problems or by an additional emotional strain on the patient[9].

We would like to present a technique that prevents occlusion[8], the quantitatively most important functional impairment of all internal drainage systems, by use of port-a-cath system without the additional emotional strain associated with percutaneous drainage[9].

### *Patients*

In the period from 1988 to 1990 a total of 28 patients with non-resectable obstructive jaundice were treated at the Department of Surgery, Hanusch Medical Center (Table 2). Endoscopic drainage as well as biliodigestive anastomosis were the principal modalities to ensure palliation (Table 3). In four patients a new drainage system was placed.

### *Indications*

Indications for palliative port-a-cath drainage were all local non-resectable stenosing processes of the supraduodenal bile duct, of the junction of the hepatic ducts, the hepatic ducts as well as compression of the junction of the hepatic ducts *ab externo* where biliodigestive anastomosis was technicaly not feasible or endoscopic drainage had failed.

### METHOD

Following laparotomy, the indication for port-a-cath drainage was established. We first performed free dissection and longitudinal incision of the bile duct peripheral to the stenosis. Via choledochotomy the tumor-induced stenosis was gradually dilated with a Charriere 17 probe. Under digital control the tip of the probe was preferably guided through the right hepatic duct, which is more easily accessible, to

**Table 2** Etiology of 28 patients with malignant non-resectable biliary obstruction

| *Diagnosis* | *Number* |
|---|---|
| Carcinoma of the gallbladder or bile duct | 14 |
| Pancreatic cancer | 7 |
| Gastric cancer | 3 |
| Carcinoma of the right colonic flexure | 2 |
| Duodenal cancer | 1 |
| Portal lymphomas of Hodgkin's disease | 1 |

**Table 3** Treatment in 28 patients with malignant non-resectable biliary obstruction

| *Treatment procedures* | *Number* |
|---|---|
| Endoscopic drainage | 10 |
| Bilio-digestive anastomosis | 7 |
| Percutaneous drainage | 4 |
| U-tube according to Terblanche (21) | 2 |
| Explorative laparotomy | 1 |
| Port-a-cath-drainage | 4 |

reach the hepatic surface, which was perforated in the direction of the anterior axillary line. The presence of diffuse liver metastases was a contraindication. Dispersed metastases localized by ultrasound or CT were identified intraoperatively. Perforation of these areas with the probe should be avoided. Intraoperative use of the ultrasonographic scanner appeared particularly beneficial. The perforated portion of a modified port-a-cath catheter was slipped over the tip of the probe and was inserted by withdrawing the probe (Figure 1). This catheter is identical with a currently available catheter designed for permanent peritoneal access (Port-a-cath$^R$, Pharmacia, Deltec Inc, St Paul, Minn, USA) with the exception of 120 holes in the distal portion instead of 80 holes in the peritoneal catheter (Custom Model 9004). The overall dimensions of the radiopaque polyurethane catheter were as follows: inside diameter 2.6 mm, outside diameter: 4.9 mm, length 48 cm, holes 1 mm in diameter.

The catheter was pulled through until the cuff was tightly applied to Glisson's capsule (Figure 2). If the position was correct, the stenosed bile was drained. In any event radiologic verification was obtained after contrast medium had been injected into the catheter. After sufficient drainage of the intrahepatic bile ducts was confirmed, the cuff was sutured to the hepatic surface and the point of passage was additionally sealed with fibrin sealant. The proximal end of the catheter was shortened so as to be located in a prepapillary position. Incision fo the papilla was unnecessary and should be avoided. The choledochotomy was then closed with running sutures(4/0), followed by dissection of a subcutaneous pouch above the right costal margin.The unperforated portion of the catheter, which was located peripheral to the cuff and emerged from the liver, was subcutaneously displaced after intercostal incision and adequate shortening. It was then connected with the port previously inserted into the pouch. The system was subsequently fixed with four sutures (Figure 3). Attention should be paid to the fact that, as with all port

**Figure 1** Catheter slipped over the tip of the probe. (*See colour plate at the back of this issue*).

**Figure 2** Catheter in regular position; cuff tightly attached to Glisson's capsule. (*See colour plate at the back of this issue*).

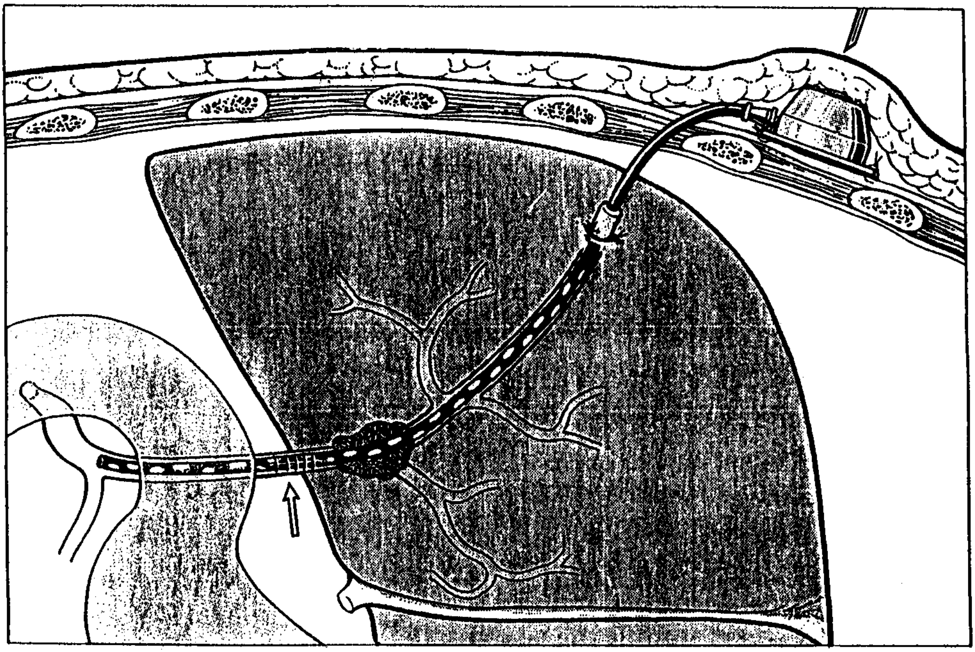

**Figure 3** Final position of the catheter after closure of choledochotomy and fixation of the port (schematic drawing).

systems, placement should not occur beneath the incision so that punctures need not be performed in the vicinity of the scar. After closure of the skin incision and the laparotomy the first transcutaneous irrigation of the system with 20 ml heparin — sodium chloride solution (1:100) took place. This was carried out in weekly intervals, after one month at 2-weekly intervals and from three months onwards at 4-weekly intervals.

### CASE REPORTS

### *Case 1*

*JC, male, 76 years*
Laparotomy for recurrence of carcinoma of the gall bladder, which was treated by simple cholecystectomy six months before (pathologic stage T1NO), revealed an egg-sized tumor invading the proximal common bile duct as well as the portal vessels. Installation of a port-a-cath drainage system was followed by normalization of hyperbilirubinemia. The patient died three weeks later of lung embolism.

### *Case 2*

*MM, female, 76 years*
In this patient a port-a-cath drainage system was implanted due to an apricot-sized carcinoma at the bifurcation of the hepatic duct, which infiltrated the pancreas and retroperitoneum as well as the lymph nodes at the hepatic porta. The patient was discharged with normal bilirubin levels. Jaundice reoccurred 4 months later. Cholangiography via the port system revealed patency, while ultrasound showed multiple liver metastases which led to the patient's death two months later.

*Case 3*

*BF, female, 77 years*
A carcinoma of the gall bladder infiltrating the liver as well as the common bile duct with diffuse peritoneal spread was diagnosed during laparotomy. In addition there were multiple metastases in both lungs.

After implantation of a port-a-cath system hyperbilirubinemia returned to normal levels. Six months later jaundice reoccurred, which led to the patient's death one month later. The autopsy revealed multiple metastases in the liver, which also occluded the catheter.

*Case 4*

*WT, female, 80 years*
The patient was admitted with jaundice and rapid deterioration of her general condition. Laparotomy revealed carcinoma of the gall bladder with metastases invading the lymph nodes and liver. In addition, there was evidence of necrotizing pancreatitis with areas of extrapancreatic necrosis. Following cholecystectomy a port-a-cath system was installed as described above. Although bilirubin levels started to fall, the patient died of pancreatitis associated with respiratory insufficiency on the 7th postoperative day.

### DISCUSSION

Due to the anatomical topography, malignant stenosis of the bile duct is often not radically resectable even at an early stage. On the other hand, the biology of hepatobiliary tumours is characterized by a markedly longer survival than with other carcinomas of the gastrointestinal tract. This is why palliative resection is a valid option in the therapy of this type of stenosis as long as morbidity and mortality are low. As resection is commonly associated with a more extensive procedure[10], at the time of surgery many patients are unsuitable for major surgery because of massive jaundice, advanced age, tumor cachexia and liver metastases.

The challenge to provide palliative drainage of incurable malignant stenosis of the bile duct has contributed to the development of a variety of surgical and non-surgical modalities. All internal drainage methods are comromised by two potential complications: dislocation, even duodenal perforation[11], and occlusion. Dohomoto reports on catheter dislocation after percutaneous transhepatic biliary drainage (PTBD)[12]. Gradual migration of the prosthesis is more common than acute dislocation. Mangegold and Jung noted catheter occlusion due to bile sediment and ascending food particles[8]. The absence of irrigability is a serious problem inherent in their use. The overall complication rate of all non-surgical biliary drainage methods is reported to range from 5 to 25%[12–15], irrespective of technical complications.

The most frequent complication is infection in the form of septic cholangitis[14]. Up to 10% of patients with transhepatic placement of catheters of long duration have been associated with life-threatening hemorrhagic complications, e.g. hemobilia[16], which appears to be less frequent with endoscopically applied stents[17]. With an incidence of 1–2% all other potential sequelae of transhepatic procedures such as biliary peritonitis, pneumo- and hematothorax as well as allergic reactions are rated as rare events[18,19]. All external drainage methods carry an additional emotional strain, as the patients are continuously reminded of their incurable condition[9]. In addition, electrolyte disturbances and hypocholia-induced diarrhea may aggravate the often seriously impaired general condition of the patient[2,20].

Use of a port-a-cath system for biliary drainage of malignant non-resectable stenosis of the extrahepatic bile duct system permits irrigation of the system as well as easy radiologic monitoring. The risk of dislocation is minimized by port fixation in a subcutaneous pouch.

Since the transpapillary approach is avoided papillary physiology is preserved. Additional advantages of the closed system are reduction of the emotional strain and prevention of fluid and electrolyte loss.

### *References*

1. Lygidakis, N.J. (1988) Kombinierte Rekonstruktion der Gallengänge und Lebergefässe bei Karzinomen der Hepatikusgabel. *Chirurg.*, **59**, 472–477
2. Hoevels, J., Lunderquist, A. and Ihse, I. (1978) Percutaneous transhepatic intubation of bile duct for combined internal-external drainage in preoperative and palliative treatment of obstructive jaundice. *Gastrointestin. Radiol.*, **3**, 23–31
3. Kremer, B. and Henne-Bruns, D. (1986) Das postoperative leberausfallskoma. *Zentralbl. Chir.*, **111**, 1166–1171
4. Calne, R.Y. (1982) Liver transplantation for liver cancer. *World J. Surg.*, **6**, 76–80
5. Klatskin, G. (1965) Adenocarcinoma of the hepatic duct at its bifurcation without the porta hepatis. An unusual tumor with distinctive clinical and pathological feature. *Am. J. Surg.*, **38**, 241–245
6. Kremer, B., Henne-burns, D., Soehendra, N., Grimm, H. and Pieper, F. (1988) Zur Problematik der chirurgischen Therapie des Hepatikusgabel-Karzinoms. *Chirurg.*, **59**, 472–477
7. Hild, P., Schwemmle, K. and Dobroschke, J. (1979) Biliodigestive Anastomosen als Palliativeingriff beim malignen extrahepatischen Verschlussikterus. *Chir. Praxis.*, **26**, 627–637
8. Manegold, B.C. and Jung, M. (1987) Endoskopisch-therapeutische Eingriffe an den Gallen-und Pankreaswegen. *Chirurg.*, **58**, 392–401
9. Pereiras, R.V., Reingold, O.J., and Hutson, D. (1978) Relief of malignant obstructive jaundice by percutaneous insertion of a permanent prosthesis in the biliary tree. *Ann. Intern. Med.*, **89**(1), 589–593
10. Lygidakis, N.J. and van der Heyde, M.N. (1988) Primary cholangiocarcinoma of the hepatic hilus. How to approach and how to choose the type of surgical management. *Acta Chir. Austr.*, **4**, 379–384
11. Coons, H.G. and Carey, P.H. (1983) Large bore long biliary endprosthesis (biliary stents) for improved drainage. *Radiology*, **148**, 89–94
12. Dohomoto, M., Schweiberer, and Kimura, K. (1982) Perkutane Drainage des Verschlussikterus. *Chirurg.*, **53**, 143–148
13. Dooley, J.S., Olney, J. and Dick, R. (1979) Non-surgical treatment of obstruction. *Lancet*, **2**, 1040–1043
14. Hanson, J., Hoevels, J. and Siemert, G. (19779) Clinical aspects of non-surgical percutaneous transhepatic bile drainage in obstructive lesions of the extrahepatic bile ducts. *Ann. Surg.*, **189**, 58–61

15. Mueller, P.R., van Sonnenberg, E. and Ferruci, Jr., J.T. (1982) Percutaneous biliary drainage: Technical and catheter related problems in 200 procedures. *Am. J. Roentgenol.*, **138**, 17–23
16. Saar, M.G., Kaufmann, S.L. and Zuidema, G.D. (1984) Management of hemobilia associated with transhepatic internal biliary drainage catheters. *Surgery*, **95**, 603–607
17. Marks, W.N., Freeny, P.C., and Ball, P.J. Endoscopic retrograde bliary drainage. *Radiology*, **152**, 357–360
18. Hrabin, B.P., Mueller, P.R. and Ferruci, Jr. J.T. (1980) Transhepatic cholangiography: Complications and use patterns of the fine needle technique. *Radiology*, **135**, 15–22
19. Rose, J.S. (1983) Invasive Radiology: Risks and patients care. Chicago: Year Book Medical Publishers, 1983
20. McPherson, G.A.D., Benjamin, I.S. and Habib, N.A. (1982) Percutaneous transhepatic drainage in obstructive jaundice: Advantages and problems. *Br. J. Surg.*, **69**, 261–264
21. Terblanche, J., and Louw, J.H. (1973) U-tube drainage in the palliative therapy of carcinoma of the main hepatic duct junction. *Surg. Clin. North Am.*,**53**, 1245–1256
22. Dick, W. and Dortenmann, J. (1965) Die Behandlung hochsitzender maligner Gallengangsstenosen. *Langenbecks Arch. Chir.*, **311**, 83–86
23. Hartenbach, W. (1972) Gallengangsendoprothesen. *Chirurg.*, **43**, 184–186
24. Yamakawa, T., Esguerra, R.T., Kaneko, H. and Fukuma, E. (1988) Percutaneous transhepatic endoprosthesis in malignant obstruction of the bile duct. *World J. Surg.*, **12**, 78–84
25. Soehendra, N. and Grimm, H. (1984) Endoskopische Drainage des Gallenganges In: Buess G., Unz, F. and Pichelmair, H. (Eds.) Endoskopische Techniken. Köln: Duetscher Ärzte-Verlag, **83**

*(Accepted by S. Bengmark 10 September 1992)*

